# Eulogium on Roux and Reminiscences of Dupuytren

**Published:** 1857-03

**Authors:** 


EULOGIUM ON ROUX, THE EMINENT SURGEON, AND REMINISCENCES OF HIS GREAT RIVAL, DUPUYTREN.—It is the duty of the perpetual secretary, perhaps we ought to say the secretary for life, of the French Academy of Medicine, to pronounce the eulogy of a deceased member at the annual meeting of that body. The office was held for many years by M. Pariset, who acquired no little reputation for the ability and, we might say, dexterity with which he could lavish praises on and barely hint defects of the departed academician. His successor, the present incumbent, M. F. Dubois (d'Amiens), already displays nearly equal tact, but with a less affluent and flowery style. At the annual public meeting of the Academy, held on the 16th of December last, in the presence of a certain number of invited persons of both sexes, M. Dubois delivered the eulogium on M. Roux. The French Journal,* from which we derive our knowledge of the address, has some piquant remarks on the occasion, which will not be without value for us on this side of the Atlantic. Credit is given to the speaker for his endeavor to exhibit all the good qualities of the head and heart, but without concealing the defects of Roux, which last prevented him from reaching the lofty renown of his predecessor at the hospital of the Hôtel Dieu, M. Dupuytren. This historic impartiality is scarcely ever attempted, still less carried out, in American biographies; and we are left, the greater part of the time, in complete ignorance of the real character, as far as regards disposition and personal habits, of the man. The biographer forgets that he is not writing merely for the gratification of a certain number of contemporaries, but for posterity, which will like to know the man as he was, irrespective of the personal predilections or prejudices to which he was subjected at the time. The French Journal points out some of the different modes of proceeding pursued by the academic orators. Some, without entirely neglecting biographical details of the hero of the hour, devote themselves, in a more especial manner, to a profound appreciation of his works. Such were, more particularly, the academic eulogiums of Arago. Others take to the anecdotal line, so that the insignificance of the personage himself is entirely lost sight of in the crowd of minor stories skilfully narrated. Although this last fashion of proceeding was not, as it could not be attempted in the case of such a person as Roux; yet neither was the other, which should consist in a complete and searching analysis of the labors of the great surgeon, carried out by M. Dubois, owing to the restraint under which he felt himself by the presence of the female portion of his auditory.

It is only on the supposition of his fear of offending the ears of the other sex, that we can account for the silence of M. Dubois on perineoraphy one of the most brilliant surgical displays of Roux. In truth, as the French reporter wittily remarks, the perpetual secretary, who, apropos of staphyloraphy, made those of both sexes who were present so clearly understand what that delicate and movable membrane at the back of the mouth was, ought not to have been deterred from some allegorical description of the perineum. Besides, who knows whether the female portion of the auditory were not dissatisfied with M. Dubois's reticence on this occasion? The auditory doubtless came, as the writer suggests, in quest of surgical emotions, a proof of which is furnished by an indiscreet feuilletonist, who relates that the ladies paid no attention to the entrance of M. Huguier and some other academicians, but that the sight of M. Ricord caused all heads to turn in his direction. "Such a licentious curiosity deserved to be punished by a long description of the suture of the perineum."

* Archiv. Gen., Janv. 1857.

A somewhat similar scene to that here alluded to, occurred at one of the sessions of the National Medical Association, when it met in Baltimore. Pains were taken by some of the members, who, on that occasion, were more good-natured than discreet, to let their friends of both sexes know that the chairman of the Committee on Periodical Medical Literature, who was well known and appreciated both as a wit and a poet, would read his report at a certain hour. Thus notified and invited, a number of amateurs of both sexes took their seats, at the time designated, in the gallery of the church in which the Association held its meetings. We do not know to what extent, or in what manner, these new auditors were affected by the report, nor how few could appreciate or relish the pungent criticisms, and ridicule of journalist defects and foibles. But we remember very well how highly amused the members of the Association, and particularly the other members of the Committee were at the embarrassment of the chairman, when he came to certain words and phrases descriptive of particular organs or particular diseases, which are seldom if ever addressed to "ears polite," and at the dexterity with which he would either omit or slur them, or substitute a somewhat equivalent but more palatable phraseology. This was the first, and it will, we believe, be the last time in which persons of the other sex will be invited, or care to show themselves, at the meeting of a Medical Association when it is engaged in the reading or discussion of professional subjects. If women should ever present themselves on such an occasion, then ought they to be saluted with full discharge of the entire description, whatever may be the subject,—disease, operation, or remedy, even to the exhibition of a sick-room apparatus, like that introduced on the French stage in the performance of *Le Malade Imaginaire.*

But, shutting our eyes on the mixed auditory at the Academy, let us revert to Roux and the perpetual secretary, M. Dubois. Notice is taken by the latter of the debut of the young aspirant for fame, by his entering himself as an army surgeon. We are told, also, of his well-known intimacy with Bichat; his first struggles with Dupuytren, and of his relations to Boyer, whose daughter he married. But we glide over these, and the notices of his subsequent life, until we reach the period of M. Roux's election as first surgeon to the Hôtel Dieu, and read the parallel drawn of him and his deceased predecessor, Dupuytren.

"He was finally at the head of French surgery. He met Dupuytren, and Dupuytren had stayed his further progress. If at times he succeeded in placing himself on the same line with Dupuytren, it was because on this same line there were many posts: wherever there was but one of these, it was Dupuytren who filled it. There could be only one chief surgeon to the king, and it was Dupuytren who obtained this signal honor; there could be only at the same time one chief surgeon to the Hôtel Dieu, and it was Dupuytren who occupied the post; and if M. Roux finally obtained it, it was because the death of his rival left him the field clear."

It was said that Roux, sensible of the weighty succession that would devolve on him, hesitated some time before he would accept it; and having once decided for himself, it is known how he was received, and the prejudices he had to encounter. And yet to see these two surgeons, Dupuytren and Roux, it would seem easy to say which of them would soonest secure the favorable opinion of young men on his side. The former had a gloomy and majestic carriage, and walked at the head of his pupils with a haughty and anxious expression of countenance; everybody was uncovered as he went along, and followed him in silence. The other showed himself with a countenance at once open, content, and smiling; greeting all, kind, obliging, and seeking by these means, to swell a somewhat noisy cortege, in whose gaiety he participated.

Dupuytren's literary education was defective, and even in his moral training there were voids which he never learned to fill up; but yet everything in him inspired respect and kept others at a distance; his speech, like his attitude and gestures, was plain, grave, and almost august. Roux aimed at elegance, and shone in other ways: it must be admitted that he indulged in repetitions and interminable terms in all his allocutions; but, at the same time, what rich reminiscences, what keenness in his perceptions—and all without preparation, without affectation, and with a charm, an easy freedom, a benevolence beyond all imitation. But as his self-constituted judges at their last "*Concours*," in which he and the shade of Dupuytren seemed to be brought in presence of each other, in the vast wards of the Hôtel Dieu, were all instinctively, as it were, hostile to M. Roux, they could only discover that the fashion of speech of the rash successor of Dupuytren was diffuse, prolix, abounding in circumlocution, embarrassed with obscurities and perpetual iterations; whilst the elocution of Dupuytren, which was lofty, exact, and sententious, was durably impressed on their memory as a classic model of correctness, method, and clearness.

On the mistaken views entertained by Roux, of the manner in which he could best efface the memory of his predecessor, viz., by continual action and movement, M. Dubois makes the following observation: "He forgot that what raised to so high a pitch the fame of his predecessor was not the number or the novelty of the operations which he performed, but rather his admirable judgment, the certainty of his diagnosis, and that rare prudence which was manifested in everything he did. It is true, that he blended with these some degree of artifice and ostentation, and that, in reality, the safety of his patients caused him perhaps less anxiety than the care of his own reputation; but as, after all, the two were inevitably linked together, these minute precautions, these deep calculations eventually turned to the profit of the sick."

After a rapid survey of the labors of Roux as a teacher, a practitioner, and a writer, M. Dubois concludes his address by a free and discriminating picture of the lamented Professor. "Three great surgeons have in a measure taken up, in our estimation, the first half of the nineteenth century: Boyer, Dupuytren, and Roux. Of these three surgeons, Roux was incontestably, as an operator, the most ingenious, the most enterprising, and the boldest; but we ought to add, that he has not always been free from censure for having carried this boldness to the extent of rashness. In his case, the qualities of the good, the true, the excellent surgeon, were summed up almost entirely in the art of operating with dexterity and grace—above all, with grace. Roux, more than any other man, had it in his power to be of service to the cause of humanity: he advanced the science, and in many points extended the limits of the art. Why did he not possess a little more of that prudence and reserve which are so necessary in the practice of surgery? He excelled in all; but never knowing when to restrain himself, he abused his powers nearly in all; not only in speech and in writing, but also in that which is the most formidable thing in the world, the art of surgery. Impatient in action, desirous of exhibiting himself with all his advantages, that is to say, with instrument in hand, he did not always take time to ascertain whether an operation was absolutely necessary, or rigorously indispensable: he only ascertained whether it was *possible*. But what operation is not possible to so skilful a surgeon?" Age could not calm his vivacity and impetuosity; and hence "that defect of order and connection which appeared, by turns, in his lectures, his writings, and his practice. How often has he not been seen, yielding to the chance inspirations of the moment, begin an operation, as he would a discourse, without knowing in advance where he would stop, or how he would finish, and himself astonished at the turns he had made and the results which he had reached.

"Roux was then a great operator, but he was so too exclusively; he was not adequately penetrated with that great and incontestable truth: that a man, to be a successful surgeon, must also be a wise physician; that what constitutes the strength and glory of surgery is, that in its study and in its practice, it is in close union with medicine."

It was our good fortune, we will not say how long ago, to walk attendance on both Dupuytren and Roux in their hospitals, the Hôtel Dieu and the Charité, for upwards of a year; visiting each of these institutions on alternate days, and hearing the clinical lectures of these two celebrated surgeons, who were then in the maturity of their powers and their fame. Roux was second surgeon to the Charité at this time, in association with his father-in-law, Boyer, who was chief surgeon. We might even speak of a certain degree of acquaintance with the Napoleon of the Hôtel Dieu, which, however, we must confess, was somewhat of the same kind as that of a boasting Gascon with a distinguished personage of whom his friend was speaking at the time. On being hard pushed to describe the kind of intercourse between himself and the dignitary in question, it was found to consist in the latter having kicked him down stairs, with an objurgatory request to leave the house. Our acquaintance with Dupuytren was not quite of so close a nature. It consisted in our expressing a hope that he would allow us to attend the hospital during his regular visits. Probably embarrassment, and a not overfluent French diction, prevented our meaning from being as fully expressed as we wished. But, be this as it may, we well remember the reply, which was much more laconic than Parisian,—"Who are you?"—uttered in a tone of impatience, and with a look of disdainful inquiry. Our rejoinder of, *Un medecin Americain*, was hardly listened to, and the great man passed on to the next bed. The request on our part was, after all, one merely of courtesy, as at that time the hospitals were open to all. In the course of the following winter, when access was not quite so easy, certain conditions were attached to the permission for French students.

Foreigners who had graduated at home, were admitted on exhibiting their diplomas.

Dupuytren's lectures were given in a theatre which was also the operating room of the hospital. They were remarkable for their clearness, and their simple and terse language, but without any pretence to methodical arrangement, and with scarcely a sprinkling of learning, neither of which, it must be confessed, are rigidly demanded in clinical teaching. The lecturer himself was seated in an open chair raised on a kind of staging, which was reached by two or three steps from the arena of the lecture-room and on the side through which light was admitted. He seldom faced his class, but, for the most part, turned to one side, with legs crossed; and as he began in rather a low voice, it cost those on whom he had turned his back, a considerable effort to hear him, although his utterance was distinct, and altogether his elocution excellent. Gradually his voice became somewhat more raised, and then, having obtained the ears of the full theatre, he commanded unfailing attention to the end.

Of this he was sure, for two reasons; First, owing to the interest inspired by his manner of handling the subject; and, secondly, to the certainty that any one of his auditors guilty of the least noise, or who disturbed others in any way, would be at once noticed, and rebuked by the lecturer, if not summarily ejected from the lecture-room. One was sure of seeing an operation, when it was performed in the lecture-room or theatre. No crowding round the table was permitted. The surgeon only allowed one, or at most two assistants to be near him; and as to the hospital dressers and resident students, *externes* and *internes*, they were ordered to be down on their haunches on the floor, or arena of the theatre. Dupuytren had a scale of sounds in his own mind, by which he measured the cries of those who were subjected to his knife on the operating table. We have seen him lay down his instrument in the midst of an operation for strangulated hernia, and tell the patient in somewhat rough speech, that he hollowed too loud—made too much noise, and that if he did not cease, or lower his tone, he would not continue the operation. The least neglect of duty, which included want of punctual attendance on the part of the house students, when the roll was called in the morning, just before the visit through the wards, was sure to meet with a harsh reprimand—such as the slave might expect from his overseer or task-master. Notwithstanding all this, when it suited his purpose, this boorish and unfeeling man, could so entirely change his whole manner and expression as to be no longer recognizable, were a screen to be interposed between him and those listening to his discourse. When, for example, he wished to allay the alarm of a child, and insure as much as possible its keeping quiet during an operation on the eye, nothing could be more soothing than his speech on the occasion, no tones more musical, more inspiring of confidence and love, than those in which he addressed *mon enfant, mon cher petit, mon mignon;* and certainly the charm at times seemed to operate as the inhalations of ether and chloroform have since done. In the city he could successfully act the same part, as we learned from the question of a lady; whether we did not find M. Dupuytren to be a most agreeable man, and remarkable for his amenity in the hospital?

All Paris, with its seemingly endless variety of manner of speech and style of address among its inhabitants, could not present so strong a contrast between any other two men, as there was between Dupuytren and Roux, as lecturers. The first, as we have seen, was slow, measured, sententious;—every period rounded, every word distinctly uttered, and carrying with it the full and desired meaning, as if cast and polished with artistic skill for the purpose. Roux, on the other hand, was voluble, and so fluent that the stream of words was unbroken by pause or cæsura, and they were so run into each other, as to fail to carry their separate meaning, so that the puzzled listener was left, half the time, to guess from the context, by his ear, now and then catching a word which compelled a little more slow syllabification. The mouth of the speaker was somewhat drawn up and projected, as when a person is about to whistle, and if, while he does this, he were to roll his tongue about, he would have no bad idea of the buccal and lingual movements of M. Roux, the intonations of whose voice were, besides, entirely deficient in clearness or resonance. How he was relished, and how appreciated as a lecturer by the French students themselves, may be understood from the remark of one of them, who asked, after we had been some months in Paris, how we got along in following the different lecturers? Our reply was, "Very well; except with M. Roux, whom we do not understand." "Ah! in truth, nor I either; none of us can make him out," was the rejoinder of our querist. And yet this man wrought his way to high position as a teacher, and kept it too. His success is, however, only one among the many examples of what talents and perseverance will procure for their possessor, even under the greatest natural disadvantages. Roux was listened to with respect, because everybody knew that he could wield the pen and the operating knife with ease, dexterity, and ability; and to the last, people could hardly believe it possible that a man, whose hand, in so marked and extraordinary a manner, and in such different ways, could execute the dictates of an active and teeming mind, should not find his organs of speech equally obedient and ready to enable him to give full utterance to his great experience, his fertile expedients, and his overflowing suggestiveness.

---

Bonpland, the veteran explorer, now in his eighty-third year, writes from Uruguay that he is about to cross the ocean, to offer his collections of botany and other specimens of natural history, to the French government; after which he will return to South America, and end his days on his plantation.

---

M. Nélaton has been lately elected to fill the vacancy in the section of surgery at the Academy, caused by the death of Gerdy, by a vote of 60 out of 78 academicians. His competitors were Denonvilliers, Hutin, Moret-Lavellee, and Bonnafont.

---

Owing to an unusual amount of reviews and original contributions, we have been compelled to omit the "Bi-Monthly Periscope" in this number. This will probably not occur again, however satisfactory it may appear to be able to offer our readers a journal full of new matter.

